# Supplementary material for: BiRNA-BERT allows efficient RNA language modeling with adaptive tokenization
Source: Commun Biol. 2025 Nov 20;8:1621. doi: 10.1038/s42003-025-08982-0 (PMC12635123; doi:10.1038/s42003-025-08982-0)
Supplement: Supplementary file 2 — Supplementary Information [file 42003_2025_8982_MOESM2_ESM.pdf]

1 Information-Theoretic Analysis of BPE vs Nucleotide Tokenization

In this section, we compare the information content of a nucleotide token and a BPE token inspired by key empirical observations in the training data. Information-theoretic analysis of biological sequences is a well-studied field of research where the key challenges include determining the prior distribution of nucleotides or k-mers, the fact that only a fraction of possible biological sequences occurs in nature and the difficulty in comparing results from biological sequences with those from linguistics due to significant differences in morphology [4, 1, 6].

Information content of a nucleotide token We consider each nucleotide in a sequence as an independent variable that carries some amount of information. We wish to quantify the maximum amount of information for each new nucleotide in a sufficiently long sequence. We can derive the per-token upper bound of the Shannon entropy of a DNA/RNA sequence as follows.

$$\begin{aligned} H(X_{NUC}) &= -\sum_{i=1}^{4} P(x_i) \log_2 P(x_i) \\ &\leq -\left(4 \cdot \frac{1}{4} \log_2 \frac{1}{4}\right) \\ &= 2 \text{ bits} \end{aligned} \tag{1}$$

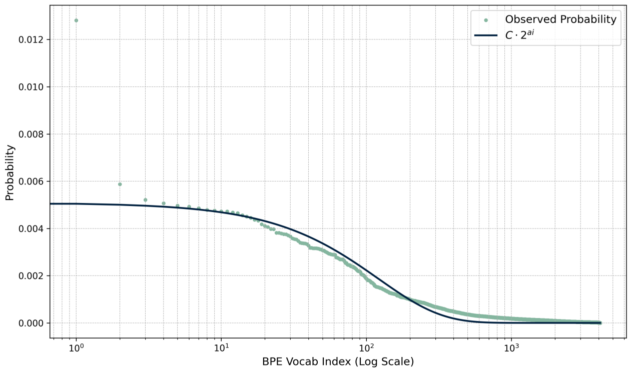

Supplementary Figure 1: Fitting the exponential function $C \cdot e^{-\alpha i}$ to the empirically observed BPE token probabilities on pretraining datasets. Since the index assigned to a token is arbitrary, we sort the tokens in an descending order of the probability/frequency and re-index tokens to represent the probability as a function of the index. We determined best-fit when $C \approx 0.005068$ and $\alpha \approx 0.011995$.

Information content of a BPE token Similar to as nucleotide tokens, we consider each BPE token in a sequence as an independent variable that carries some amount of information. Let the size of the vocabulary be N. On our pretraining datasets, we observe that the frequency of the BPE tokens is exponentially distributed, and as a result, the probability of a token can modeled by an exponential function as follows.

$$P(x_i) = \frac{C}{2^{ai}} \tag{2}$$

Since the index assigned to a token is arbitrary, tokens can be sorted by descending probability and reindexed without issue. Under this formulation:

$$\begin{aligned}
&\sum_{i=1}^{N} \frac{C}{2^{ai}} = C \sum_{i=1}^{N} \frac{1}{2^{ai}} = 1 \\
&\Leftrightarrow \frac{C}{2^a} \cdot \frac{1 - \left(\frac{1}{2^a}\right)^N}{1 - \frac{1}{2^a}} = 1 \\
&\Leftrightarrow \frac{C\left(1 - \frac{1}{2^{aN}}\right)}{2^a - 1} = 1 \\
&\Leftrightarrow C = \frac{2^a - 1}{1 - \frac{1}{2^{aN}}}
\end{aligned} \tag{3}$$

When the vocabulary size N is large, we can approximate $C \approx 2^a - 1$ and $a \approx log_2(C+1)$. Now we can derive a general expression for the entropy of BPE

tokens as,

$$
\begin{aligned}
H(X_{BPE}) &= -\sum_{i}^{N} P(x_i)\log_2 P(x_i) \\
&= -\sum_{i=1}^{N} \frac{C}{2^{ai}} \log_2\left(\frac{C}{2^{ai}}\right) \\
&= -\sum_{i=1}^{N} \frac{C}{2^{ai}} (\log_2 C - ai) \\
&= -\log_2 C \sum_{i=1}^{N} \frac{C}{2^{ai}} + aC\sum_{i=1}^{N} \frac{i}{2^{ai}}, \\
&= -\log_2 C + aC\sum_{i=1}^{N} \frac{i}{2^{ai}}, \\
&\approx -\log_2 C + aC\frac{2^a}{(2^a-1)^2}\ , \qquad \text{When } N \text{ is large} \\
&= -\log_2 C + \log_2(C+1)C\frac{C+1}{(C)^2} \\
&= -\log_2 C + \log_2(C+1)\left(\frac{C+1}{C}\right) \\
&= \log_2\left(\frac{(C+1)^{(C+1)/C}}{C}\right).
\end{aligned} \tag{4}
$$

If the weighted average length of a BPE token is $\bar{L} = \sum_{i=1}^{N} P(x_i)\text{len}(x_i)$, the average character-level entropy of BPE representation of a sequence will be $\hat{H}(X_{BPE}) = \frac{H(X_{BPE})}{\bar{L}}$. Since nucleotides are one character each, the per-character entropy is $\hat{H}(X_{NUC}) = H(X_{NUC})$. The BPE tokenization will lead to less entropy if,

$$
\begin{aligned}
&\frac{\hat{H}(X_{BPE})}{\hat{H}(X_{NUC})} < 1 \\
\Rightarrow &\frac{H(X_{BPE})}{\bar{L} \times H(X_{NUC})} < 1 \\
\Rightarrow &\log_2\left(\frac{(C+1)^{(C+1)/C}}{C}\right) < 2 \times \bar{L} \\
\Rightarrow &\frac{(C+1)^{(C+1)/C}}{C} < 4^{\bar{L}}.
\end{aligned} \tag{5}
$$

When $C << 1$, we can approximate $(C+1) \approx 1$. Then the inequality in Equation 5 can be further simplified as

$$
\frac{1}{C} < 4^{\bar{L}} \Rightarrow C > 4^{-\bar{L}}.
$$

Empirical Entropy Ratio On our pretraining data mixture, we determine that $P(A) \approx 0.2726$, $P(T) \approx 0.2144$, $P(C) \approx 0.26642$, $P(G) \approx 0.2465$, and averge BPE token length $\bar{L} \approx 6.0768$. This yields the empirical entropy of nucleotide tokens $H_e(X_{NUC}) \approx 1.9939$ bits. As shown in Figure 1, the empirical value of C is 0.005086 when determined on 33 million sequences of our pretraining dataset.

Plugging in this value in Eqn. 4 yields $H_e(X_{BPE}) \approx 9.1044$ bits. Therefore, the empirical per-character entropy ratio is

$$\frac{\hat{H}_e(X_{BPE})}{\hat{H}_e(X_{NUC})} = \frac{H_e(X_{BPE})}{\bar{L} \times H_e(X_{NUC})} \approx \frac{9.1044}{6.0768 \times 1.9939} \approx 0.7514 < 1.$$

The empirical per-character entropy ratio of 0.7514 indicates that the BPE tokenization technique effectively compresses the input sequence. Although compressed information is likely more difficult for language models to process, it is well-compensated by the ability to process sequences up to 6 times longer than the original input with the same GPU memory constraints. This also partially explains why we observed BPE underperforming their NUC counterparts on short-sequence downstream tasks from an Information-theoretic perspective.

Therefore, BPE tokenization is essentially a trade-off between information compression and computational efficiency, which BiRNA-BERT can dynamically adjust depending on the hardware constraints and sequence length.

Here, we assume tokens are independent and identically distributed random variables (i.i.d) to approximate the information content of NUC and BPE sequences. In reality, the information content of non-i.i.d sequences is much lower than Shannon Entropy due to the correlation between nearby symbols. Language entropy [5] and Kolmogorov Complexity [2] take symbol correlation and order into account but are generally intractable.

Optimal Vocabulary Size From Figure 1, it is evident that after the vocabulary size exceeds 2000 tokens, the frequency of new tokens significantly decreases, resulting in very rare appearances of those tokens. Therefore, increasing the vocabulary size beyond 4096 tokens offers diminishing returns in terms of encoding the sequence, particularly in long RNA sequences. Moreover, recent benchmarking studies on RNA language models, such as the work presented in BEACON [3], demonstrate that for k-mer tokenization, which is relevant to our context, the optimal value for k is 6. For $k = 6$, the corresponding vocabulary size is $4^6 = 4096$, which aligns with our choice of vocabulary size for BiRNA-BERT. While we did not empirically test the optimal vocabulary size for BPE in RNA language models in our manuscript, both the information-theoretic analysis and the findings from recent benchmarking studies support that a vocabulary size of 4096 is indeed an optimal strategy for BiRNA-BERT.

2 Supplementary Figures

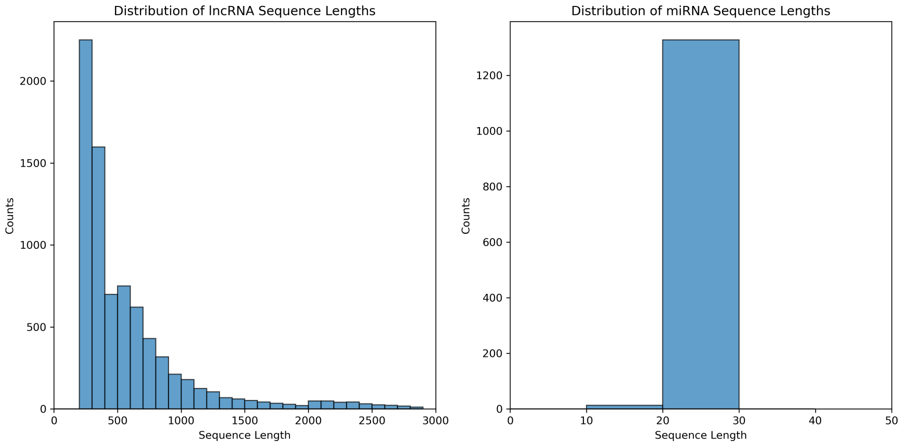

Supplementary Figure 2: Distribution of sequence lengths in the lncRNA and miRNA datasets.

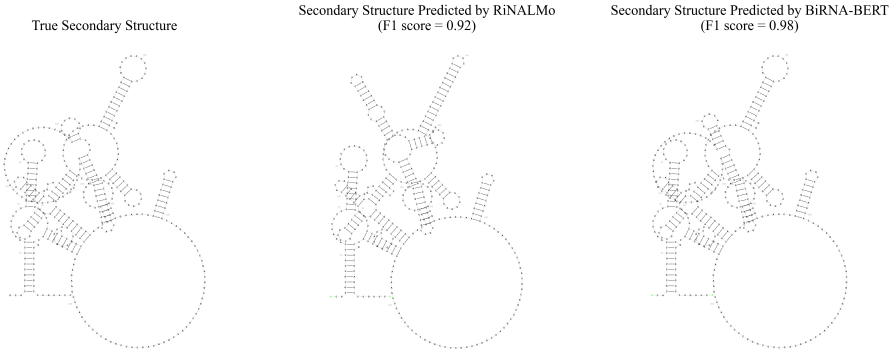

Supplementary Figure 3: Visualization of RNA secondary structure prediction task for the longest sequence in the testing dataset by RiNALMo and BiRNA-BERT.

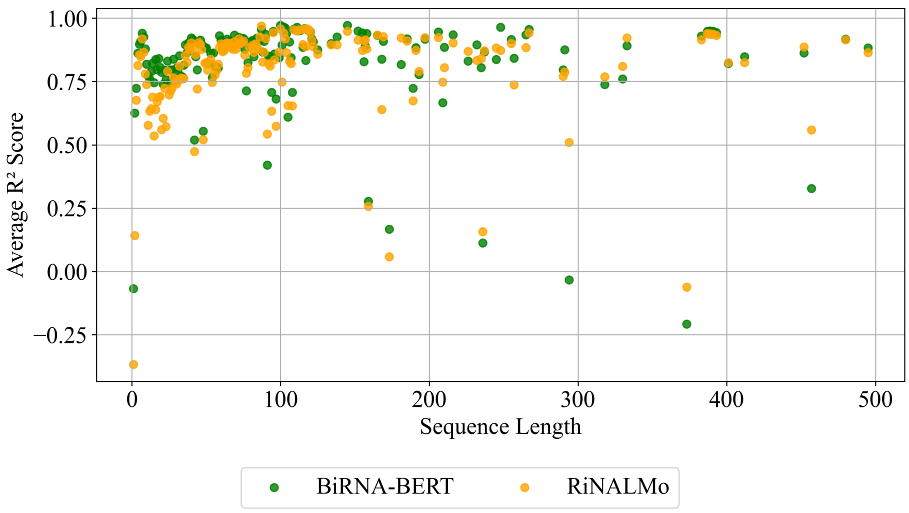

Supplementary Figure 4: Lengthwise performance (average R2 score) comparison of BiRNA-BERT and RiNALMo for RNA 3D distance map prediction.

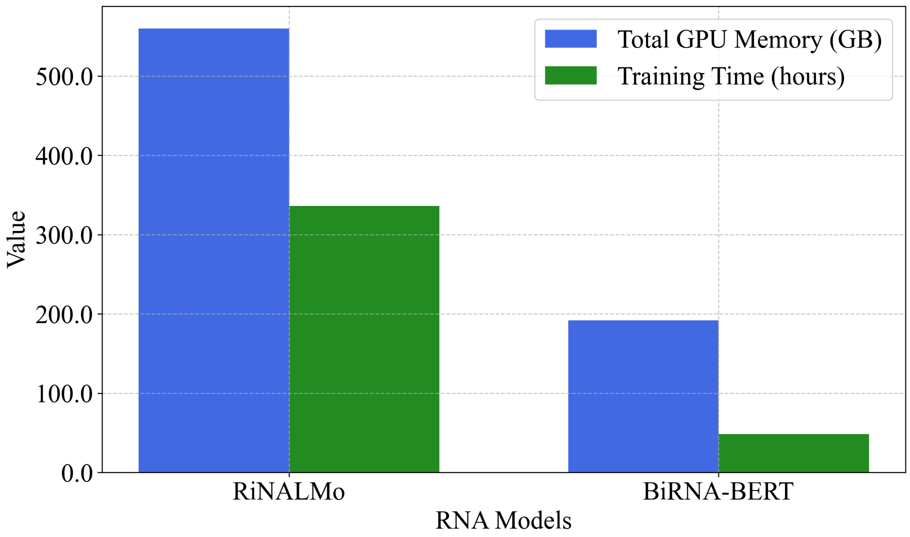

Supplementary Figure 5: Comparison of GPU Memory Usage and Training Time for Different Models.

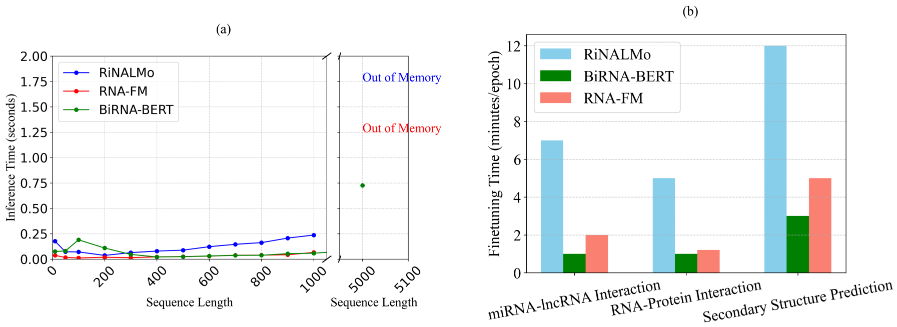

Supplementary Figure 6: Comparison of inference and finetuning time for different RNA language models. (a) Inference time for a single sequence across different lengths. For sequences with length over 1024 nucleotides, RiNALMo and RNA-FM provides out of memory error, while BiRNA-BERT provides embedding even for such length with consistency (b) Finetuning time per epoch for three different downstream tasks. In each case, BiRNA-BERT requires significantly lower training time.

3 Supplementary Tables

Supplementary Table 1: Summary of the benchmark dataset for the miRNA-lncRNA interaction prediction task.

**Benchmarks of RNA–RNA interactions**		**No. of miRNAs**	**No. of lncRNAs**	**No. of molecule pairs**
**Arabidopsis thaliana (ATH)**	Interacting Pairs	331	2014	2500
	Non-interacting Pairs	266	1964	2500
**Glycine max (GMA)**	Interacting Pairs	401	1770	2500
	Non-interacting Pairs	542	171	2500
**Medicago truncatula (MTR)**	Interacting Pairs	335	1986	2500
	Non-interacting Pairs	424	2442	2500

Supplementary Table 2: Dataset Specification for RNA-Protein Interaction Prediction

**Dataset**	**Train**	**Valid**	**Test**
AATF	26283	6571	8214
ABCF1	28768	7193	8991
AGGF1	76800	19200	24000
AKAP1	76800	19200	24000
AARS	76800	19200	24000

Supplementary Table 3: Dataset Specification for RNA N6-methyladenosine Prediction

**Species**	**Tissue**	**Training Pos**	**Training Neg**	**Test Pos**	**Test Neg**
**Human**	Liver	2634	2634	2634	2634
	Brain	2302	2303	1150	1150
	Kidney	2287	2287	1144	1143
**Mouse**	Brain	4013	4012	4013	4012
	Kidney	1977	1976	1976	1977
	Liver	2066	2067	2066	2067
**Rat**	Brain	1176	1176	1176	1176
	Kidney	1716	1716	1716	1716
	Liver	881	881	881	881

Model	Central Masking		15% Random Masking	
	**Perplexity ↓**	**Recovery ↑**	**Perplexity ↓**	**Recovery ↑**
**BPE-Based Models**				
BiRNA-BERT (BPE)	2.14	32%	1.27	27.0%
**NUC-Based Models**				
BiRNA-BERT (NUC)	1.42	89%	1.50	87.0%
RNA-FM	1.43	87%	1.55	84.0%
RiNALMo	1.42	90%	1.50	88.9%

Supplementary Table 4: Perplexity (↓) and token recovery accuracy (↑) of RNA language models under 15% random masking and central masking schemes. Lower perplexity and higher recovery indicate better model performance.

References

[1] Christoph Adami. Information theory in molecular biology. *Physics of Life Reviews*, 1(1):3–22, 2004.

[2] A.N. Kolmogorov. On tables of random numbers. *Theoretical Computer Science*, 207(2):387–395, 1998.

[3] Yuchen Ren, Zhiyuan Chen, Lifeng Qiao, Hongtai Jing, Yuchen Cai, Sheng Xu, Peng Ye, Xinzhu Ma, Siqi Sun, Hongliang Yan, et al. Beacon: Benchmark for comprehensive rna tasks and language models. *Advances in Neural Information Processing Systems*, 37:92891–92921, 2024.

[4] Armin O Schmitt and Hanspeter Herzel. Estimating the entropy of dna sequences. *Journal of theoretical biology*, 188(3):369–377, 1997.

[5] Claude Elwood Shannon. A mathematical theory of communication. *The Bell system technical journal*, 27(3):379–423, 1948.

[6] Susana Vinga. Information theory applications for biological sequence analysis. *Briefings in bioinformatics*, 15(3):376–389, 2014.
